# Validation of heart failure quality of life tool and usage to predict all-cause mortality in acute heart failure in Uganda: the Mbarara heart failure registry (MAHFER)

**DOI:** 10.1186/s12872-018-0959-1

**Published:** 2018-12-12

**Authors:** Samson Okello, Fardous Charles Abeya, Boniface Amanee Elias Lumori, Suzan Joan Akello, Christopher Charles Moore, Brian H. Annex, Andrew J. Buda

**Affiliations:** 1 Department of Internal Medicine, Mbarara University of Science and Technology, P. O Box 1410, Mbarara, Uganda; 2 Department of Educational Foundations and Psychology, Mbarara University of Science and Technology, Mbarara, Uganda; 3 Department of Medicine, University of Virginia Health System, Charlottesville, Virginia, USA; 4 Bernard Lown Scholars in Cardiovascular Health Program, Department of Global Health and Populations, Harvard T.H Chan School of Public Health, Boston, MA USA

**Keywords:** Acute heart failure, All-cause mortality, Kansas City cardiomyopathy questionnaire, 36-item short form health survey, Sub-Saharan Africa

## Abstract

**Background:**

The health-related quality of life (HRQoL) is an important treatment goal that could serve as low-cost prognostication tool in resource poor settings.

We sought to validate the Kansas City Cardiomyopathy Questionnaire (KCCQ) and evaluate its use as a predictor of 3 months all-cause mortality among heart failure participants in rural Uganda.

**Methods:**

The Mbarara Heart Failure Registry Cohort study observes heart failure patients during hospital stay and in the community in rural Uganda. Participants completed health failure evaluations and HRQoL questionnaires at enrollment, 1 and 3 months of follow-up. We used Cronbach’s alpha coefficients to define internal consistency, intraclass correlation coefficients as a reliability coefficient, and Cox proportional hazard models to predict the risk of 3 months all-cause mortality.

**Results:**

Among the 195 participants who completed HRQoL questionnaires, the mean age was 52 (standard deviation (SD) 21.4) years, 68% were women and 29% reported history of hypertension. The KCCQ had excellent internal consistency (87% Cronbach alpha) but poor reliability. Independent predictors of all-cause mortality within 3 months included: worse overall KCCQ score (Adjusted Hazard ratio (AHR) 2.9, 95% confidence interval (CI) 1.1, 8.1), highest asset ownership (AHR 3.6, 95% CI 1.2, 10.8), alcoholic drinks per sitting (AHR per 1 drink 1.4, 95% CI 1.0, 1.9), New York Heart Association (NYHA) functional class IV heart failure (AHR 2.6, 95% CI 1.3, 5.4), estimated glomerular filtration rate (eGFR) 30 to 59 ml/min/1.73 m^2^ (AHR 3.4, 95% CI 1.1, 10.8), and eGFR less than 15 ml/min/1.73 m^2^ (AHR 2.7, 95% CI 1.0, 7.1), each 1 pg/mL increase in Brain Natriuretic Peptide (BNP) (AHR, 1.0, 95% CI 1.0, 1.0), and each 1 ng/mL increase in Creatine-Kinase MB isomer (CKMB) (AHR 1.0, 95% CI 1.0, 1.1).

**Conclusion:**

The KCCQ showed excellent internal consistency. Worse overall KCCQ score, highest asset ownership, increasing alcoholic drink per sitting, NYHA class IV, decreased estimated glomerular filtration rate, BNP, and CKMB predicted all-cause mortality at 3 months. The KCCQ could be an additional low-cost tool to aid in the prognostication of acute heart failure patients.

**Electronic supplementary material:**

The online version of this article (10.1186/s12872-018-0959-1) contains supplementary material, which is available to authorized users.

## Background

The assessment of health-related quality of life (HRQoL) in heart failure patients provides perspectives on how heart failure affects their well-being, an index which cannot be obtained directly from clinical measurements [1]. Improving patients’ HRQoL is increasingly accepted as an important treatment goal [2, 3] and as such HRQoL assessment could serve as a low-cost method to aid in the prognostication of heart failure patients.

The Kansas City Cardiomyopathy Questionnaire (KCCQ) is a widely used heart failure specific HRQoL measure which has been translated and culturally adapted [4], with demonstrated good psychometric properties in numerous studies [5–8]. Although it is important to capture the health status of heart failure afflicted individuals using a heart failure specific health status tool like the KCCQ [7], the use of a generic health status measure, such as the Medical Outcomes Study 36-item Short Form Health Survey (SF-36) in the same individuals, may also aid in understanding the community preferences [9]. This would allow comparison of results within the larger context of other populations and treatment approaches [10].

However, before HRQoL measures are rolled-out in any setting, an assessment of the psychometric properties are key especially in sub-Saharan Africa where heart failure is increasingly prevalent with high fatality rates [11, 12]. Thus, the present study aimed to, 1) compare the internal consistency of the KCCQ and SF-36 HRQoL measures, and 2) to evaluate the use of the KCCQ, a heart failure specific HRQoL measure, as a predictor of all-cause mortality 3 months following an acute heart failure hospitalization episode. We hypothesized that KCCQ has a higher internal consistency compared to the SF-36, and that a poor heart failure related quality of life as measured by the KCCQ scale predicts all-cause mortality among heart failure patients in rural Uganda.

## Methods

### Study population

Participants were selected from the Mbarara Heart Failure Registry (MAHFER), a longitudinal study on heart failure outcomes in southwestern Uganda, conducted at the Mbarara Regional Referral Hospital (ClinicalTrials.gov Identifier: NCT02721030) described elsewhere [13]. Briefly, patients aged 13 years or greater from the hospital catchment area (estimated 8 million people) were consecutively enrolled into MAHFER from June 2015 to March 2017. In addition to the attending physicians’ diagnosis of heart failure, patients were screened for inclusion based on the clinical symptoms and signs of heart failure as per established criteria [14] except for testing of natriuretic peptide (BNP > 35 pg/ml or NT-proBNP > 125 pg/ml) which are not available for routine clinical care as is the case in most resource poor settings. In the present study, the tests were performed after enrollment to guide clinical care and not for diagnosis. Patients with an acute exacerbation of chronic kidney disease, chronic obstructive pulmonary disease, or acute liver disease with no features of heart failure were excluded. Participants were followed daily during hospitalization and every month post discharge at the cardiology outpatient clinic until 3 months or death, whichever comes first.

### Data collection

We administered standardized questionnaires to collect data on participant demographics, past medical history (i.e., cardiovascular risk factors and co-morbid conditions), prior hospitalizations and discharge medications, New York Heart Association (NYHA) functional class, review of symptoms, vital signs and physical exam, acute cardiovascular-related and non-cardiovascular therapies, hospital course (i.e. in-hospital worsening HF and other adverse events), and outpatient course. The questionnaire also captured information on household asset ownership, smoking history (age of starting, duration and intensity of smoking and efforts to quit), alcohol intake using the Alcohol Use Disorders Identification Test (AUDIT-C) questionnaire [15], history of diagnosis and/or management of cardiovascular disease and its risk factors (hypertension, diabetes mellitus).

Pre-specified data collection was done during all days during the index hospital stay following enrollment and monthly outpatient visits following hospital discharge or until death within 3 months of enrollment. At each outpatient visit, a study nurse obtained updated medical and medication history, NYHA functional class, review of symptoms, vital signs, medication adherence, and interval events including hospitalizations.

At enrollment on the day of hospitalization, a trained study nurse performed the following measurements: plasma glucose, blood urea nitrogen, creatinine, sodium, potassium, Brain natriuretic peptide (BNP), and Creatine-Kinase (MB isomer) using a point of care i-Stat Analyzer (Abbott Point of Care, Princeton, New Jersey, USA).

Left ventricular ejection fractions (LVEF) were measured by transthoracic echocardiography (HD7 XE Diagnostic ultrasound system, China) and LVEF was categorized as reduced (≤ 40%), midrange (41 to 49%), or preserved (≥ 50%) [14].

### Health related quality of life measurements

On the second day of hospitalization, a bilingual study nurse administered translated or English language paper-based questionnaires of the Medical Outcomes Study 36-Item Short Form Health Survey (SF-36) and the Kansas City Cardiomyopathy Questionnaire (KCCQ) [7] to all participants. The Kansas City Cardiomyopathy Questionnaire (KCCQ) is a 23-item questionnaire used to quantify physical limitation, symptom stability, symptom frequency, symptom burden, self-efficacy, quality of life, and social limitation each measured using a Likert scale and two summary subscales: the overall KCCQ score and Clinical summary score. The scores for all subscales range from 0 to 100, with higher scores indicating better health status [7]. The SF-36 questionnaire covers physical functioning, physical limitation, emotional limitation, bodily pain, general health, mental health, social functioning, energy fatigue, and physical health each measured using a Likert scale. The scores for these components range from 0 to 100, with higher scores indicating better health status [16, 17].

### Mortality

For the current analysis, the outcome of interest was 3 months all-cause mortality because the KCCQ and SF-36 were assessed at enrollment, 1 and 3 months of follow-up. All-cause mortality was determined by a combination of medical record review for hospitalized participants and 2 weekly telephone calls to participants’ family to obtain date and circumstances leading to death. At enrollment, participants were asked for contact information for at least 3 family members or next of kin in case the participant could not be contacted. Participants were considered lost to follow up if neither the participant nor the 3 contacts were unreachable/inaccessible at least on 3 different occasions on 3 consecutive days. All-cause mortality was classified as in-hospital if it occurred during hospitalization including during subsequent re-hospitalizations, or community death if the participant died at home.

### Statistical analysis

We generated an asset index score – a measure of socioeconomic status – based on household characteristics, utilities, and ownership of durable assets using principle component analysis [18]. Participants were divided into asset index quintiles: poorest, poor, average, rich, and richest.

The subscales of the KCCQ and SF-36 questionnaires were scored (in percentages) as previously described [7, 19]. We then created a composite overall KCCQ score by adding subscales: Physical limitation, total symptom score, quality of life, and social limitation. The overall KCCQ score was then categorized into worst score (0 to 24%), poor (25 to 49%), fair (50 to 74%), and good (75 to 100%).

Descriptive statistics were used to summarize characteristics of the entire cohort using mean (SD) for continuous normally distributed parameters such as age, median (Interquartile range, IQR) for skewed variables e.g., length of hospital stay, etc. Proportions were used to describe the ceiling and floor effects (highest and lowest scores on each instrument) at baseline (time 0) so as to evaluate the extent to which extreme scores at each assessment limited the capacity of the scales to detect further change in health-related quality of life. Floor or ceiling effects were present when at least 15% of participant scored the lowest or highest possible score, respectively.

We conducted two principal analyses. First, we tested the assumption of unidimensionality of the SF-36 and KCCQ using confirmatory factor analysis and then calculated Cronbach’s alpha coefficients to define internal consistency and intraclass correlation coefficients (ICC) as a reliability coefficient of the KCCQ subscales and composite overall scale. We measured the internal consistency of the baseline measurements of SF-36 subscales and composites. Cronbach’s alpha coefficients > 0.80 were considered indicative of high internal consistency and an ICC of 0.70 was considered as a minimum standard for good reliability [20, 21].

Second, we evaluated the KCCQ score as a predictor of all-cause death within 3 months using a Cox proportional-hazards model because there was negligible competing risk (2% lost to follow up). The time of follow-up was calculated from the study enrollment date (time 0 also called baseline) until either death, loss to follow-up, or the end of 3-month follow-up, whichever came first. Cox proportional hazard models were fitted to predict the risk of all-cause death adjusted for a priori selected variables: age, gender, asset ownership index, behavioral factors (smoking, alcohol, medication adherence), comorbid conditions (hypertension, diabetes mellitus, kidney dysfunction, & HIV), New York Heart Association (NYHA) functional class, and left ventricular ejection fraction. We fit prediction models and compared discrimination indices (i.e. C-index) of a KCCQ only model (Model1), Model 2 including others variables (described above) excluding KCCQ, Model 3 consisting of variables and KCCQ, and Model 4 with variables, KCCQ, BNP, and CKMB.

Two-sided z-tests were used to assess for differences in survival by strata of potential predictors and a *p*-value of < 0.05 was considered to be statistically significant. All analyses were performed with STATA**®** Statistical Software version 15 (StataCorp LP, College Station, Texas, USA).

## Results

We screened 396 consecutive patients for eligibility and enrolled 217 participants between June 1, 2015, and March 28, 2017. Of those enrolled 2 participants with primary kidney disease with no evidence of heart failure were excluded. For the current analysis, 195 participants with HRQoL questionnaires responses (at enrollment, 1 and 3 months of follow-up) were used after exclusion of 16 participants: 8 (3.7%) participants died before administration of the KCCQ and SF-36 health status questionnaires and 8 (3.7%) participants completed health status questionnaires once.

The mean age of the cohort was 52 (standard deviation, SD 21.4) years similar to other studies from sub-Saharan Africa [22, 23]; 132 (67.7%) were women because of their better health seeking behavior [24, 25], 56 (28.7%) had a history of hypertension, 15 (7.7%) had a history of diabetes mellitus, 16 (8.2%) were infected with human immunodeficiency virus (HIV), and 109 (55.9%) had a NYHA class of IV (Table 1). At enrollment, the etiologies of heart failure included: hypertensive heart disease in 42 (21.5%), dilated cardiomyopathies in 39 (20.0%) aischemic heart disease in 6 (3.1%), and of participants. There was no difference in baseline characteristics of participants included for this analysis and those lost to follow up or those who did not complete all questionnaires.

**Table 1:** Baseline characteristics of HF patients, MAHFER study

	*N* = 195
Characteristic	
Age, mean (SD), years	52 (21.4)
Women, *n* (%)	132 (67.7)
Asset index quintiles, *n* (%)	
Poorest	33 (16.9)
Poorer	32 (16.4)
Average	34 (17.4)
Rich	38 (19.5)
Richest	34 (12.3)
Missing	24 (12.3)
Highest education level attained, *n* (%)	
None	73 (37.4)
Primary	105 (53.9)
Secondary & Tertiary	17 (8.7)
Smoking history, *n* (%)	
Never smoked	153 (78.5)
Former smoker	24 (12.3)
Current smoker	18 (9.2)
Alcohol use history, *n* (%)	
Never	19 (9.7)
Non-hazardous	172 (88.2)
Hazardous	4 (2.1)
Comorbid diseases	
Hypertension, *n* (%)	56 (28.7)
Diabetes mellitus, *n* (%)	15 (7.7)
HIV infection, *n* (%)	16 (8.2)
None	108 (55.4)
Self-reported poor medication adherence	115 (59)
Aetiology of Heart failure	
Hypertensive heart disease	42 (21.5)
Dilated cardiomyopathies	39 (20.0)
Ischemic heart disease	6 (3.1)
Unknown	108 (55.4)
NYHA^¥^ functional class at enrollment	
NYHA class I, *n* (%)	0 (0)
NYHA class II, *n* (%)	1 (0.5)
NYHA class III, *n* (%)	85 (43.6)
NYHA class IV, *n* (%)	109 (55.9)
Left ventricular ejection fraction (LVEF)	
LVEF^*^, mean (SD)	41 (12.9)
Medication during hospital stay*, *n* (%)	
Furosemide	178 (91.3)
ACEI/ARB^∧^	54 (27.7)
Digoxin	45 (23.1)
Dobutamine	20 (10.3)
Blood test	
Blood urea nitrogen (mg/dL), mean (SD)	59.6 (65.4)
Serum Creatinine (mg/dL), mean (SD)	2.0 (2.7)
Serum sodium (mmol/L), mean (SD)	132 (15.8)
BNP^∞^(pg/mL), mean (SD)	2164 (1762)
CKMB^€^ (ng/mL), mean (SD)	4.3 (10.2)

*SD* standard deviation, *NYHA*^*¥*^ New York Heart Association, LVEF^*^ Left ventricular Ejection fraction, *HIV* Human Immunodeficiency virus, *ACEI/ARB*^*∧*^ Angiotensin Converting Enzyme Inhibitor/Angiotensin Receptor Blocker, *BNP*^*∞*^ Brain Natriuretic Peptide, *CKMB*^*€*^ Creatine-Kinase (MB isomer)

*Most participants took multiple medications thus the percentages of medications add up to more than 100%

We found statistically significant changes in mean scores at the 1st and 3rd months time points for all KCCQ subscales except total symptom score. However, the overall KCCQ score intra-class correlation coefficients (ICC) for all KCCQ subscales were low ranging from 0.08 (0.01, 0.56) for symptom burden to 0.45 (0.29, 0.62) for self-efficacy (Table 2).

**Table 2:** Longitudinal mean changes and intraclass correlation coefficients (ICC) of KCCQ subscales, MAHFER study

HQOL measure	Mean (SD)	Mean change vs. Baseline (SE)	*P*-value trend	ICC (95% CI)
KCCQ				
Physical limitation				0.16 (0.05, 0.41)
Baseline	12.6 (18.9)	–	–	
Month 1	41.9 (28.9)	−29.4 (3.0)	< 0.001	
Month 3	41.2 (25.9)	−28.8 (3.5)	< 0.001	
Symptom stability				0.24 (0.08, 0.53)
Baseline	51.5 (21.1)	–	–	
Month 1	48.5 (20.7)	2.9 (2.9)	0.450	
Month 3	46.1 (21.7)	5.4 (3.7)	0.142	
Symptom frequency				0.14 (0.03, 0.45)
Baseline	38.4 (22.9)	–	–	
Month 1	47.3 (30.9)	−9.1 (3.5)	0.046	
Month 3	52.9 (32.0)	−14.5 (4.2)	0.003	
Symptom burden				0.08 (0.01, 0.56)
Baseline	40.6 (24.7)	–	–	
Month 1	42.7 (33.3)	−8.7 (3.8)	0.046	
Month 3	52.4 (35.3)	−18.5 (4.7)	0.001	
Total symptom score				0.11 (0.02, 0.48)
Baseline	39.5 (23.8)	–	–	
Month 1	45.0 (31.9)	−8.8 (3.6)	0.046	
Month 3	52.7 (33.4)	−16.5 (4.4)	0.002	
Self-efficacy				0.45 (0.29, 0.62)
Baseline	58.7 (21.7)	–	–	
Month 1	31.5 (24.3)	27.2 (3.1)	< 0.001	
Month 3	25.0 (24.4)	33.6 (3.8)	< 0.001	
Quality of life				0.14 (0.03, 0.44)
Baseline	23.1 (16.1)	–	–	
Month 1	14.5 (22.7)	8.7 (2.5)	0.005	
Month 3	13.1 (22.2)	10.2 (2.9)	0.024	
Social limitation				0.13 (0.03, 0.41)
Baseline	11.9 (18.7)	–	–	
Month 1	13.0 (28.9)	−1.2 (3.0)	0.008	
Month 3	13.8 (26.9)	−2.0 (3.5)	0.905	
Overall summary score				0.13 (0.03, 0.43)
Baseline	21.8 (15.8)	–	–	
Month 1	28.6 (23.4)	−7.7 (2.5)	0.008	
Month 3	30.2 (21.4)	−9.3 (2.9)	0.002	
Clinical summary score				0.16 (0.04, 0.44)
Baseline	26.0 (18.6)	–	–	
Month 1	43.4 (25.3)	−19.2 (2.8)	< 0.001	
Month 3	46.9 (24.9)	−22.6 (3.4)	< 0.001	

*HQOL* Health related quality of life, *KCCQ* Kansas City Cardiomyopathy Questionnaire, *SD* Standard deviation, *SE* Standard Error, *ICC* Intraclass Correlation Coefficient

At baseline, the KCCQ unlike SF-36 scale, displayed negligible floor or ceiling effects and both had acceptable distribution of scores (Table 3). Flooring effects were evident for the KCCQ subscales of physical limitation and social limitation and for the SF-36 the physical limitation and emotional limitation subscales. None of the KCCQ subscales manifested ceiling effects in contrast to the bodily pain, mental health, social functioning, and physical health subscales of the SF-36. (Table 3) The floor and ceiling effects of SF-36 show that clustering of participants responses at the extremes might miss the disease effect [26].

**Table 3:** Baseline ceiling, floor effects, and Cronbach’s alpha coefficients of the KCCQ and SF-36 scales, MAHFER study

HQOL measure	Mean (SD)	Median (IQR)	% Floor effect	% Ceiling effect	Inter-item correlation	Cronbach’s alpha coefficients
KCCQ						
Physical limitation	12.6 (18.9)	0 (0, 25)	59.9	2.5	0.46	0.83
Symptom stability	51.5 (21.1)	55 (30, 70)	0.1	8.7	0.51	0.86
Symptom frequency	38.4 (22.9)	31 (25, 50)	3.5	1.0	0.39	0.79
Symptom burden	40.6 (24.7)	33 (25, 50)	3.5	3.0	0.40	0.80
Total symptom score	39.5 (23.8)	33 (25, 50)	3.5	1.0	0.48	0.85
Self-efficacy	58.7 (21.7)	75 (50, 75)	23.5	10.4	0.47	0.84
Quality of life	23.1 (16.1)	25 (12, 25)	14.8	1.0	0.45	0.83
Social limitation	11.9 (18.7)	0 (0, 25)	61.7	2.0	0.99	0.99
Clinical summary score	26.0 (18.6)	20 (12, 38)	3.55	0.5	0.58	0.87
SF-36						
Physical functioning	23.6 (18.5)	20 (5, 40)	0.5	3.7	0.30	0.78
Physical limitation	4.4 (15.5)	0 (0, 0)	89.1	3.6	0.28	0.75
Emotional limitation	9.6 (14.4)	12.5 (0, 12)	48.7	3.7	0.27	0.75
Bodily pain	24.5 (19.9)	10 (10, 42)	7.5	23.7	0.29	0.76
General health	39.8 (10.7)	40 (30, 45)	2.1	4.7	0.35	0.81
Mental health	32.3 (9.3)	32 (26, 38)	1.0	99	0.29	0.77
Social functioning	40.9 (20.1)	37 (25, 62)	23.3	92.2	0.31	0.79
Energy fatigue	38.5 (15.3)	35 (30, 50)	0.5	3.6	0.34	0.81
Physical health	22.7 (10.8)	21 (16, 26)	1.3	26.1	0.23	0.71

The internal consistency of the overall KCCQ scale was excellent (Cronbach’s alpha of 87%). The highest single-item sensitivity of Cronbach’s alpha internal consistency was reported for Symptom stability (86%) on the KCCQ scale and general health (81%) on the SF-36 scale. The Cronbach’s alpha for each KCCQ subscale ranged from 79 to 86% when one subscale was excluded. Overall, the SF-36 had comparably good internal consistency with Cronbach’s alpha coefficient of 79%, though the Cronbach’s alpha of subscales varied between 71 and 81% (Table 3).

At the first month follow-up visit point, 35 participants had died. Among the 160 surviving participants, 4 (1.9%) were lost to follow up, and 75 (47%) completed another set of KCCQ questionnaires. At 3 months, a total of 82 participants had died and among the 113 surviving participants, 45 (40%) completed the last set of KCCQ questionnaires. By the end of the 3rd month of follow up, 82 (42%) had died (50 participants died in the community and 32 died in hospital). We found statistically significant differences in the KCCQ overall summary and clinical summary scores upon comparison of baseline KCCQ scores among those who died and those who survived until 3 months (Additional file 1: Table S1). On the contrary, a similar comparison for SF-36 showed a difference for only bodily pain.

Independent predictors of all-cause mortality within 3 months included: worse overall KCCQ score (AHR 2.9, 95% CI 1.1, 8.1), highest asset ownership (AHR 3.6, 95% CI 1.2, 10.8), alcoholic drinks per sitting (AHR per 1 drink 1.4, 95% CI 1.0, 1.9), New York Heart Association (NYHA) functional class IV heart failure (AHR 2.6, 95% CI 1.3, 5.4) (Table 4). Other predictors of mortality included: estimated glomerular filtration rate (eGFR) 30 to 59 ml/min/1.73 m^2^ (AHR 3.4, 95% CI 1.1, 10.8), and eGFR less than 15 ml/min/1.73 m^2^ (AHR 2.7, 95% CI 1.0, 7.1) and point of care blood tests Brain Natriuretic Peptide (BNP) (each 1 pg/mL increase, AHR, 1.0, 95% CI 1.0, 1.0), and Creatine-Kinase MB isomer (CKMB) (each 1 ng/mL increase, AHR 1.0, 95% CI 1.0, 1.1). (Table 4).

**Table 4:** Predictors of 3-months all-cause mortality among acute heart failure participants in rural Uganda, MAHFER study

Characteristic	Model 1 AHR (95% CI)	Model 2 AHR (95% CI)	Model 3 AHR (95% CI)	Model 4 AHR (95% CI)
Age, each year increase		1.0 (0.9, 1.0)	1.0 (0.9, 1.0)	1.0 (0.9, 1.0)
Men		0.8 (0.4, 1.7)	0.8 (0.4, 1.7)	0.8 (0.3, 1.6)
Women		Ref	Ref	Ref
Overall KCCQ score				
Poor (KCCQ 25 to 49%)	Ref		Ref	Ref
Fair (KCCQ score 50 to 74%)	0.6 (0.2, 1.6)		1.4 (0.4, 13)	1.3 (0.1, 12.9)
Worst (KCCQ score 0 to 24%)	1.4 (0.8, 2.2)		2.9 (1.1, 8.0)	2.9 (1.1, 8.1)*
Asset index, quintiles				
Poorest		1.4 (0.5, 3.8)	1.1 (0.4, 3.0)	0.9 (0.3, 2.5)
Poor		2.6 (1.0, 6.7)*	1.8 (0.6, 5.0)	1.6 (0.6, 4.6)
Average		Ref	Ref	Ref
Rich		0.8 (0.2, 2.5)	0.7 (0.2, 2.4)	0.8 (0.3, 2.8)
Richest		2.8 (1.0, 7.5)*	3.5 (1.2, 9.8)*	3.6 (1.2, 10.8)*
Prior Heart failure hospitalizations		1.3 (0.9, 1.8)	1.3 (0.9, 1.8)	1.3 (0.9, 1.8)
Self-reported good medication adherence		Ref	Ref	Ref
Self-reported poor medication adherence		1.6 (0.8, 3.1)	1.8 (0.9, 3.7)	1.8 (0.9, 3.7)
Smoking status				
Never		Ref	Ref	Ref
Former		1.1 (0.4, 2.7)	0.9 (0.4, 2.2)	0.9 (0.3, 2.4)
Current		2.4 (0.3, 20.9)	2.7 (0.3, 24.4)	2.1 (0.2, 19.9)
Alcoholic drinks per sitting (each 1 increase)		1.4 (1.0, 1.9)*	1.4 (1.0, 2.0)*	1.4 (1.0, 1.9)*
History of hypertension		0.5 (0.2, 0.9)*	0.5 (0.2, 0.9)*	0.4 (0.2, 0.9)*
History of diabetes mellitus		2.5 (0.9, 7.0)	2.3 (0.8, 6.3)	2.3 (0.8, 6.4)
History of HIV infection		0.9 (0.4, 2.0)	1.1 (0.5, 2.3)	1.0 (0.4, 2.3)
NYHA functional class				
Class III		Ref	Ref	Ref
Class IV		2.7 (1.4, 5.2)**	2.8 (1.4, 5.7)**	2.6 (1.3, 5.4)**
Left ventricular ejection fraction				
Reduced (≤ 40%)		1.2 (0.5, 2.9)	1.1 (0.4, 2.8)	1.2 (0.4, 3.0)
Middle range (41–49%)		Ref	Ref	Ref
Preserved (≥ 50%)		1.2 (0.4, 3.6)	1.0 (0.3, 3.0)	0.9 (0.3, 2.9)
Renal function eGFR^##^ (ml/min/1.73 m^2^)				
≥ 90		Ref	Ref	Ref
60–89		0.6 (0.3, 1.6)	0.6 (0.2, 1.5)	0.7 (0.3, 1.7)
30–59		2.4 (0.8, 7.1)	2.6 (0.8, 7.7)	3.4 (1.1, 10.8)*
15–29		2.1 (0.6, 7.6)	1.5 (0.4, 5.6)	1.7 (0.4, 6.8)
< 15		3.4 (1.4, 8.3)**	3.0 (1.3, 7.3)*	2.7 (1.0, 7.1)*
Cardiac biomarkers				
BNP^∞^, each 1 pg/mL increase				1.0 (1.0, 1.0)*
CKMB^€^, each 1 ng/mL increase				1.0 (1.0, 1.1)*

*AHR* Adjusted Hazard Ratio, *KCCQ* Kansas City Cardiomyopathy Questionnaire, *NYHA* New York Heart Association functional class, *eGFR*^*##*^ Estimated glomerular filtration rate, *BNP* Brain Natriuretic Peptide; CKMB^€^: Creatine-Kinase (MB isomer)

*p < 0.05; **p < 0.01; ***p < 0.001

Note: 1. There were no participants with good (KCCQ score 75 to 100%) overall KCCQ score

2. The estimated glomerular filtration rate (eGFR) was estimated using the Chronic Kidney Disease Epidemiology Collaboration (CKD-EPI) equation for blacks stratified by gender (mL/min/1.73 m^2^)

3. The C-statistics for models increased as more variables were added by model i.e., Model 1 (56.6%), Model 2 (73.1%), Model 3 (74.8%), and Model 4 (75.7%)

## Discussion

In this study of acute heart failure patients in rural Uganda, we found worse overall KCCQ score predicted mortality within 3 months of hospitalization with heart failure. Though KCCQ clinical summary score has been shown to predict survival in chronic heart failure [27], to the best of our knowledge, ours is the first report demonstrating the overall KCCQ score as an independent predictor of survival in patients with acute heart failure. In fact, our finding of a higher Cronbach alpha coefficient for the KCCQ in contrast to SF-36 indicates that for the KCCQ a large portion of the variance in the test is attributable to disease factors thereby reflecting the “true” differences in disease severity among patients [28]. This result demonstrates that the KCCQ is a better measure of heart failure specific health status than the SF-36. The KCCQ hones in on what is especially salient for heart failure and thus sensitive to clinically relevant differences or changes in health status unlike the SF-36 – a generic health status measure – which casts a broad net across different facets of health [28] and as such does not isolate the dimension of greatest interest, thereby masking the true disease effect. In fact, our study population had severe heart failure with New York Heart Association (NYHA) functional class III and IV. Also, the time intervals between the repeated administrations might have been too long to capture clinical change from time of hospitalization or too short to differentiate clinical changes between hospitalizations.

Our finding of a low prevalence of ischemic heart disease, compared to developed countries, is similar to results from a recent meta analysis of studies from the region [22]. This could be due to the low burden of atherosclerotic risk factors (e.g., smoking) in this cohort. However, the burden of ischemic heart disease is expected to increase with westernization and rise cardiovascular risk factors [29] as demonstrated in the current and other studies [30] and by the high prevalence of hypertensive heart disease. It is not surprising that cardiomyopathies contributed a fraction of heart failure etiologies since dilated cardiomyopathy is endemic [31].

In addition, our results reaffirm other predictors of mortality following hospitalization with heart failure including increasing alcoholic drinks per sitting, New York Heart Association (NYHA) functional class IV heart failure [32, 33]. This is likely explained by the rampant late presentation with advanced disease [12, 34]. Deteriorating renal function stages III and V predicted mortality [35, 36] but not stage IV as there were fewer participants in this stage. Also, we found increases in BNP and CKMB predicted all-cause mortality as has been established by previous studies [37–39]. However, there are no similar studies from sub-Sahara Africa for comparison of these results.

In addition, we found being in the group with the highest asset ownership (richest) compared to those with fair asset ownership predicted mortality. This could be a reflection of the high burden of CV risk factors among the richest group. In fact, the majority of former smokers and all those with ischemic heart disease were those with the richest asset ownership. Future studies evaluating these relationships should be pursued.

There are several features of this observational study which merit further comment. The MAHFER is a single center observational experience of heart failure patients in a resource poor setting that links the initial acute hospitalization with subsequent clinical events with unprecedented follow-up duration. Some data were based on self-reports and may be prone to reporting bias; however, self-rated health related quality of life measures such as KCCQ and SF-36 are ubiquitously used, have been shown to be strongly associated with morbidity and mortality, and are increasingly being applied as health indicators. Any bias introduced by non-response is likely to have underestimated the effect of KCCQ score on mortality. As a result, we conclude that the SF-36 scale is not able to differentiate participants by heart failure severity and therefore is not useful as a predictor of heart failure related mortality. Finally, we were unable to determine the actual causes of death due to limitations with autopsy in the study setting.

## Conclusion

In conclusion, worse overall KCCQ score, highest asset ownership, increasing alcoholic drink per sitting, NYHA class IV, decreased estimated glomerular filtration rate, BNP, and CKMB predicted all-cause mortality at 3 months. We encourage the use of the KCCQ not only for assessing health-related quality of life but also as an additional tool to predict mortality in acute heart failure patients in resource-limited settings.

## Additional file

Additional file 1: **Table S1.** Comparison means of KCCQ and SF-36 subscale according to events’ occurrence, MAHFER study. (DOCX 16 kb)

## Abbreviations

AHR: Adjusted Hazard ratio
AUDIT-C: Alcohol Use Disorders Identification Test questionnaire
BNP: Brain Natriuretic Peptide
CI: Confidence interval
CKMB: Creatine-Kinase MB isomer
eGFR: Estimated glomerular filtration rate
HIV: Human Immunodeficiency virus
HRQoL: Health-related quality of life
ICC: Intraclass correlation coefficients
IQR: Interquartile range
KCCQ: Kansas City Cardiomyopathy Questionnaire
LVEF: Left ventricular ejection fractions
MAHFER: Mbarara Heart Failure Registry
NYHA: New York Heart Association functional class
SD: Standard deviation
SF-36: Medical Outcomes Study 36-item Short Form Health Survey
