# Supplementary material for: Validation of heart failure quality of life tool and usage to predict all-cause mortality in acute heart failure in Uganda: the Mbarara heart failure registry (MAHFER)
Source: BMC Cardiovasc Disord. 2018 Dec 12;18:232. doi: 10.1186/s12872-018-0959-1 (PMC6291962; doi:10.1186/s12872-018-0959-1)
Supplement: Supplementary file 1 — Table S1. Comparison means of KCCQ and SF-36 subscale according to events’ occurrence, MAHFER study. (DOCX 16 kb) [file 12872_2018_959_MOESM1_ESM.docx]

Table S1 Comparison means of KCCQ and SF-36 subscale according to events’ occurrence, MAHFER study

| **HQOL measure** | **Alive (n=109)** | **Dead (n=82)** | **p-value** |
| --- | --- | --- | --- |
| KCCQ | Mean (se) | Mean (se) |  |
| Physical limitation | 15.3 (1.9) | 9.0 (1.9) | 0.025 |
| Symptom stability | 51.4 (2.7) | 52.0 (2.7) | 0.759 |
| Symptom frequency | 42.2 (2.3) | 33.9 (2.3) | 0.014 |
| Symptom burden | 44.5 (2.5) | 36.1 (2.5) | 0.021 |
| Total symptom score | 43.3 (2.4) | 35.0 (2.4) | 0.017 |
| Self-efficacy | 57.6 (2.2) | 59.5 (2.5) | 0.580 |
| Quality of life | 27.3 (1.6) | 18.7 (1.5) | <0.001 |
| Social limitation | 15.1 (2.0) | 7.9 (1.7) | 0.009 |
| Overall summary score | 25.2 (1.6) | 17.7 (1.5) | 0.001 |
| Clinical summary score | 29.3 (1.9) | 22.0 (1.9) | 0.007 |
| SF-36 |  |  |  |
| Physical functioning | 24.9 (1.8) | 21.7 (2.1) | 0.238 |
| Physical limitation | 4.0 (1.1) | 4.9 (2.2) | 0.676 |
| Emotional limitation | 9.9 (1.2) | 9.8 (2.0) | 0.977 |
| Bodily pain | 27.9 (2.1) | 20.8 (2.4) | 0.027 |
| General health | 40.1 (0.9) | 39.2 (1.3) | 0.564 |
| Mental health | 32.9 (1.1) | 31.5 (1.6) | 0.454 |
| Social functioning | 44.1 (2.7) | 37.2 (2.8) | 0.086 |
| Vitality | 38.5 (1.3) | 39.2 (2.0) | 0.779 |

Note: 4 participants were lost to follow up
